# An immunogenic cell death-related gene expression signature in predicting prognosis of pancreatic ductal adenocarcinoma

**DOI:** 10.1186/s12864-024-10106-7

**Published:** 2024-02-23

**Authors:** Xiaobo Wang, Tianxiang Ren, Chuting Liao, Yong Xie, Jing Cao

**Affiliations:** 1 Department of General Surgery, the Second Xiangya Hospital of Central South University, Changsha, China; 2 Zhongnan Hospital of Wuhan University, Wuhan, China; 3 Department of Breast Surgery, Xiangya Hospital of Central South University, Changsha, China

**Keywords:** Immunogenic cell death, Pancreatic ductal adenocarcinoma, Tumor microenvironment, Immune infiltration, Prognosis model

## Abstract

**Background:**

Immunogenic cell death (ICD) has been identified as regulated cell death, which is sufficient to activate the adaptive immune response. This study aimed to research ICD-related genes and create a gene model to predict pancreatic ductal adenocarcinoma (PAAD) patients’ prognosis.

**Methods:**

The RNA sequencing and clinical data were downloaded from the TGCA and GEO databases. The PAAD samples were classified into two subtypes based on the expression levels of ICD-related genes using consensus clustering. Based on the differentially expressed genes (DEGs), a prognostic scoring model was constructed using LASSO regression and Cox regression, and the scoring model was used to predict the prognosis of PAAD patients. Moreover, colony formation assay was performed to confirm the prognostic value of those genes.

**Results:**

We identified two ICD cluster by consensus clustering, and found that the the ICD-high group was closely associated with immune-hot phenotype, favorable clinical outcomes. We established an ICD-related prognostic model which can predict the prognosis of pancreatic ductal adenocarcinoma. Moreover, depletion of NT5E, ATG5, FOXP3, and IFNG inhibited the colony formation ability of pancreatic cancer cell.

**Conclusion:**

We identified a novel classification for PAAD based on the expression of ICD-related genes, which may provide a potential strategy for therapeutics against PAAD.

## Introduction

Pancreatic ductal adenocarcinoma(PAAD) is known for its aggressive nature and poor overall 5-year survival rate of approximately 12% [1]. Over the past two decades, its incidence has doubled [2]. Currently, surgical resection is the only potentially curative option for PAAD [3]. However, only a small percentage of patients are eligible for surgery, as the majority are diagnosed at advanced stages [4]. Even after radical surgery, the 5-year survival rate remains low at 25% due to local recurrence or distant metastasis [5]. Given this challenging scenario, there is an urgent need for new biomarkers to predict and improve the prognosis of PAAD patients.

Immunogenic cell death (ICD) has been identified as a regulated cell death process capable of activating the adaptive immune response [6]. Over the past decade, extensive research has been conducted to elucidate the mechanisms underlying ICD and its ability to trigger an anticancer immune response [7–9]. These studies have shown that ICD induction ultimately contributes to long-lasting antitumor immunity within the tumor microenvironment. Pancreatic cancer evades immune surveillance by exhibiting low immunogenicity and undergoing cell death through tolerogenic pathways [8]. However, ICD can timely release various damage-associated molecular patterns (DAMPs), thereby stimulating an anti-tumor adaptive immune response.

The objective of this study was to identify ICD-associated biomarkers and develop an ICD-related risk model that predicts the immune microenvironment and prognosis in PAAD.

## Materials and methods

### Datasets

The RNA-seq transcriptome information, the somatic mutations, copy number variation data and matching clinicopathological data of PAAD patients were acquired from Cancer Genome Atlas (TCGA) database (https://portal.gdc.cancer.gov/). GSE57495 from the GEO database was used for the validation (https://www.ncbi.nlm.nih.gov/geo/query/acc.cgi?acc=GSE57495).

### Consensus clustering

To classify patients into distinct molecular subtypes, we performed unsupervised consensus clustering of ICD-related genes by utilizing the R package “ConsensusClusterPlus”. Subsequently, we assessed the ideal cluster numbers between k = 2–10, and this process was replicated 1,000 times to guarantee that the results would be stable. The pheatmap tool in R was utilized to create a cluster map.

### Identification of differentially expressed genes (DEGs) between ICD-high and ICD-low subtypes

The differential mRNAs expression was assessed utilizing the Limma package (version: 3.40.2) of R software. In order to rectify false-positive TCGA data, the adjusted P values were examined. The screening criteria for mRNAs differential expression determined as adjusted *P* < 0.05 and| fold change| >2 [10].

### Functional enrichment analysis

To explore the potential mechanism underlying ICD low and high cohorts involved in PAAD, we performed the Kyoto Encyclopedia of Genes and Genomes (KEGG) [11–13] analysis using “ClusterProfiler” R package. The q-value and p-value thresholds of < 0.05 were set as the thresholds.

Then 50 gene sets of cancer hallmark-related pathways from the Gene Set Enrichment Analysis (GSEA) database were collected and GSEA was conducted to assess whether there were considerable variations in the set of genes expressed between the ICD low and high cohorts in the enrichment of the MSigDB Collection (c2.cp.kegg.v7.4.symbols.gmt) [14].

### Characterization of the immune Landscape between two ICD subgroups

To identify immune characteristics of 502 HNSCC samples, their expression data were loaded into CIBERSORT (https://cibersort.stanford.edu/) and repeated 1000 times to determine the relative percentage of 22 immune cell types [15]. Then, we compared the relative percentage of 22 immune cell types between the two ICD subgroups, and the results are presented in a landscape map.

### Construction of the ICD-Related risk signature

Least Absolute Shrinkage and Selection Operator Regression (LASSO) is a form of penalized regression that can be used to screen variables from high-dimensional data to build a prognostic model. In this study, we filtered the significant ICD-related genes from PAAD specimens and performed the optimum survival cutoff analysis. Then, we used the LASSO method in a Cox regression model to pick out the most useful prognostic genes. After that, an ICD-related scoring system for PAAD patients was established by the combination of the expression of genes and the estimated Cox regression coefficient: ICD-related risk score =∑ (coefficient of gene* expression of a gene).

And then, according to the best cutoff risk score, patients with PAAD were divided into high-risk group and low-risk group and then subjected to the Kaplan-Meier (KM) survival analysis.

### RNA interference and colony formation

The small interfering RNAs were purchased from ThermoFisher: SiNT5E (11,636), SiATG5 (137,766), SiFOXP3(108,456), SiIFNG (144,586). The manufacturer’s recommendations were followed for siRNA transfection when using Lipofectamine 2000 (Invitrogen, Carlsbad, CA, USA). PANC-1 cells were seeded to be 70–90% confluent at transfection. For the colony formation assay, the cells were treated with the indicated siRNAs for 24 h, digested, and seeded into six-well plates at a density of 1,000 cells per well. After 14 days of incubation, the cells were fixed with 4% paraformaldehyde and visualized by 0.5% crystal violet staining. Each plate was washed by 3 thorough immersions in pure water and then scanned by camera.

## Results

### Identification of ICD-associated subtypes based on consensus clustering in PAAD

**Fig. 1:**
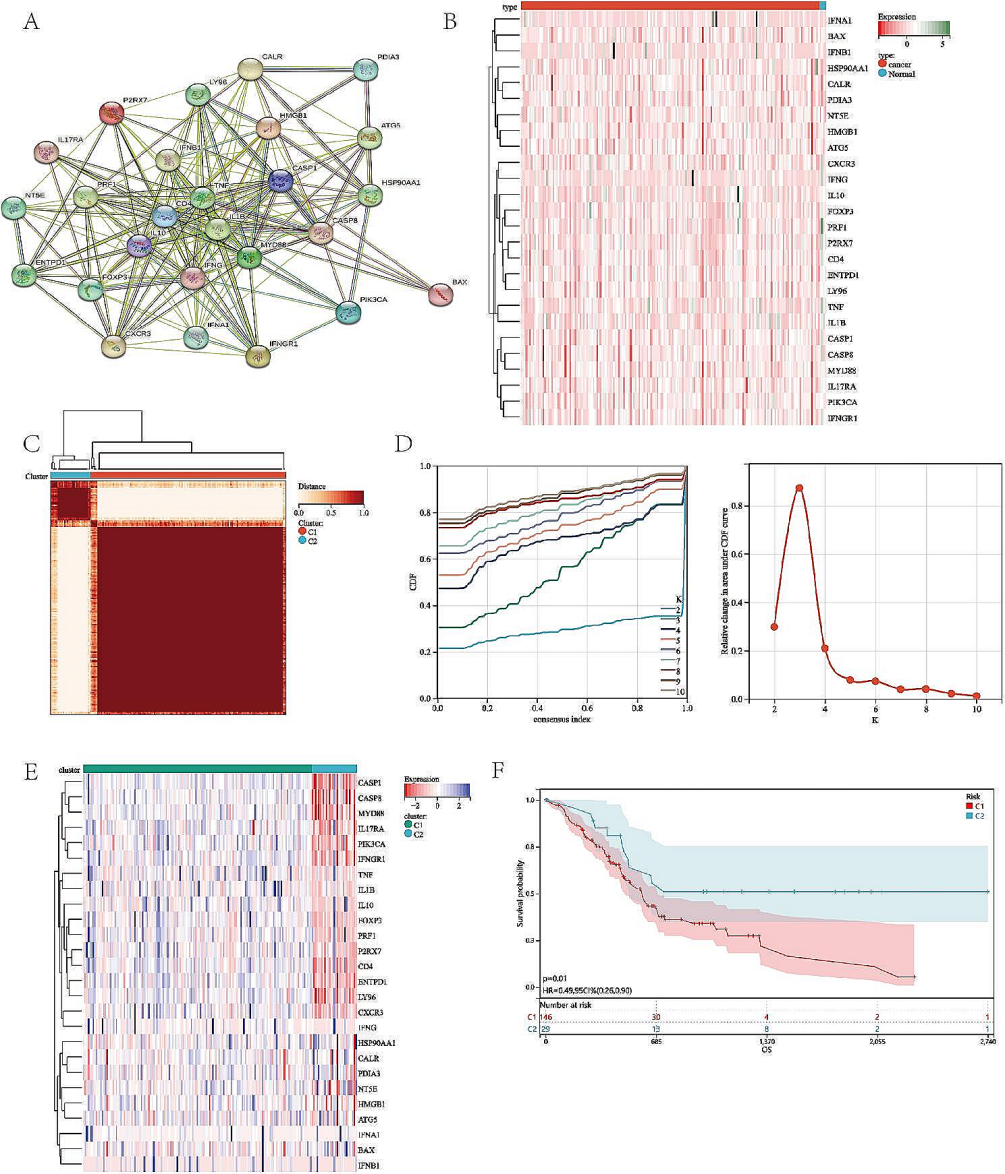
Identification of ICD-associated subtypes based on consensus clustering in PAAD. (A) Protein–protein interactions among the ICD-related genes. (B) Heatmap of the ICD-related genes expression among normal samples and PAAD samples in TCGA database. (C) Consensus clustering matrix (k = 2) of PAAD samples. (D) Consensus clustering cumulative distribution function (CDF) with k = 2 to 10. (E) Heatmap of ICD-related genes expression between different subtypes. (F) Kaplan–Meier curves of OS in different subtypes

The ICD-related genes have been previously summarized by Abhishek [15], which including CALR, ENTPD1, NT5E, HMGB1, HSP90AA1, ATG5, BAX, CASP8, PDIA3, PIK3CA, CXCR3, IFNA1, IFNB1, IL10, TNF, CASP1, IL1B, P2RX7, LY96, MYD88, CD4, FOXP3, IFNG, IFNGR1, IL17RA, and PRF1. Firstly, to depict the connections of these ICD-related genes, we utilized the STRING database to conduct protein-protein interaction (PPI) network. As shown in Fig. 1A, there were complex interactions between these genes. Next, we analyzed the expression of ICD-related genes in normal samples and pancreatic cancer samples in The Cancer Genome Atlas (TCGA). Importantly, we found that most of ICD-related genes were overexpressed in pancreatic cancer samples (Fig. 1B).

Next, to further explore the profile and characteristics of ICD-related genes in pancreatic cancer, consensus cluster analysis was performed to determine the ICD-associated clusters in PAAD patient samples. To obtain the optimal clustering number (k value), we calculated the consistency coefficient. Figure 1D showed the cumulative distribution function (CDF) curve of consensus clustering for k = 2 to 10 and we found that k = 2 was a preferable selection for sorting the entire cohort into Clusters 1 (*n* = 146) and Clusters 2 (*n* = 29) (Fig. 1C, D). As a results, the TCGA cohort was grouped into two clusters, C1 and C2. Next, we analysed the expression level of ICD-related genes between C1 and C2 and found that Clusters 1 revealed higher ICD-related genes expression level (Fig. 1E). Moreover, the Kaplan-Meier survival analysis exhibited different clinical results. In general, Cluster 2 with lower ICD-related genes had a superior overall survival (log-rank test, *p* = 0.01) among pancreatic cancer patients (Fig. 1F).

### Identification of differentially expressed genes and signal pathways in different ICD subtypes in PAAD

**Fig. 2:**
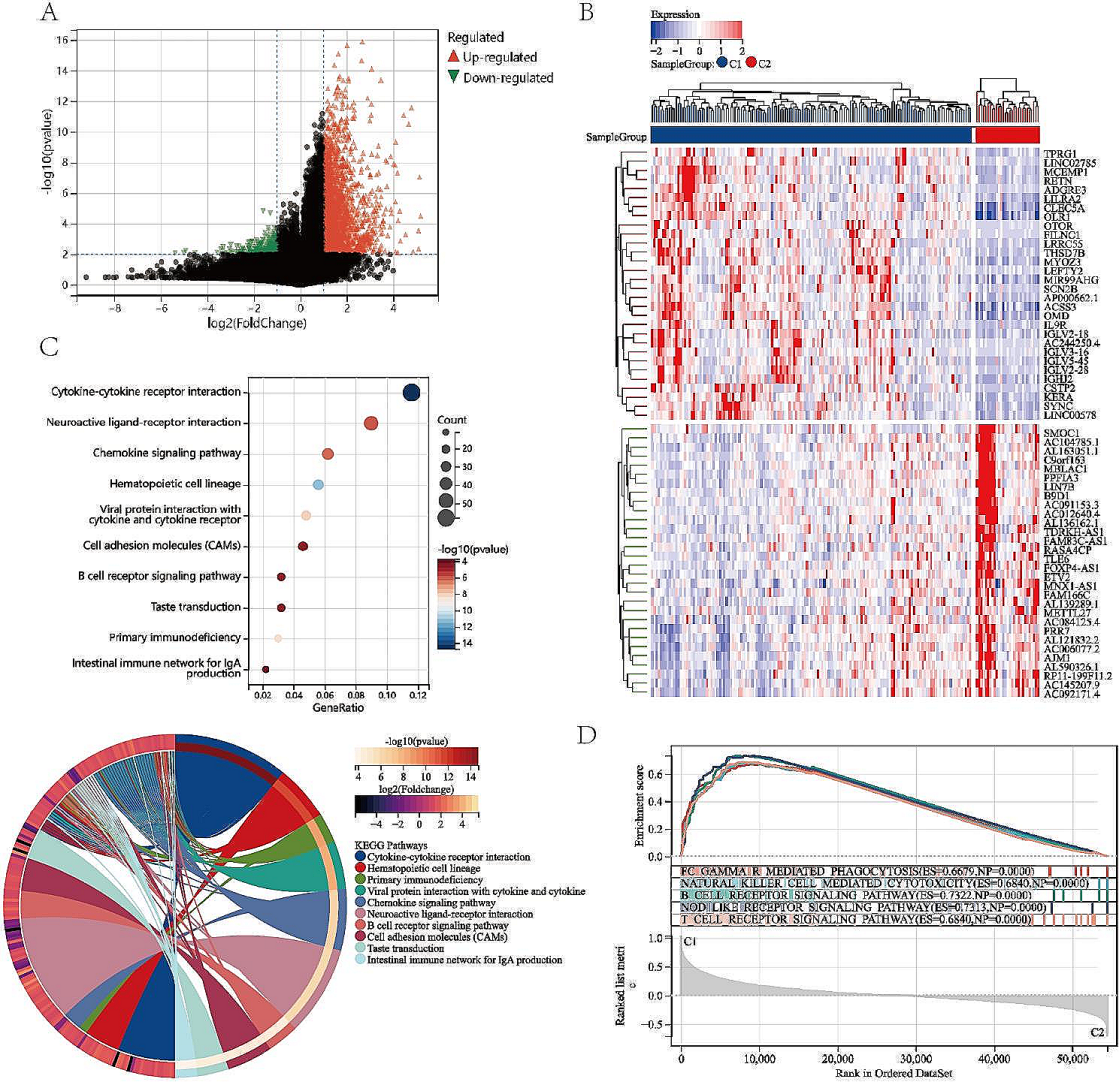
Identification of Differentially Expressed Genes and signal pathways in different ICD subtypes in PAAD. (A) Volcano plot of the distribution of DEGs quantified between different subtypes. (B) Heatmap of the top 30 up-regualted and down-regulated DEGs in different subtypes. (C) Enrichment analyses of KEGG pipelines for DEGs. (D) GSEA analysis of the significant pathway between ICD-high and ICD-low subtypes

Given the two ICD-associated subtypes indicated different clinical results, we identified the key Differentially Expressed Genes (DEGs) between Clusters 1 and Clusters 2. The characteristics of PAAD patients are list in Table 1 and no significant difference was found between C1 and C2 in age, gender, T staging, N staging, and stage. After data prepossessing and analysis, we identified an aggregate of 3,008 dysregulated genes, including 2,686 upregulated genes and 322 downregulated genes (Fig. 2A, B). Moreover, we performed functional enrichment analysis to investigate the biological behavior of these DEGs. The KEGG pathway enrichment analysis implied that these upregulated genes in the ICD high subtype were frequently enriched in cytokine-cytokine receptor interaction, chemokine signaling pathway, viral protein interaction with cytokine and cytokine receptor, B cell receptor signaling pathway primary immunodeficiency, and intestinal immune network for IgA production, most of which were involved in activities of immunity (Fig. 2C).

**Table 1:** Clinical characteristics of Cluster 1 and Cluster 2

Characteristics	C1	C2	p Value
	146	29	
**Age**			
Median	64.94 (57–73)	64.29 (57–72)	0.77
**Gender**			
Male	80	16	0.97
Female	66	13	
**T staging**			
T1	6	1	0.23
T2	17	7	
T3	120	19	
T4	3	0	
**N staging**			
N0	38	12	0.06
N1	106	15	
**Stage**			
I	14	7	0.09
II	124	20	
II	3	0	
IV	4	0	

Furthermore, the GSEA analysis were performed to identify the associated signaling pathways activated in the ICD-high subtype. We found that the immune pathways, including Fc gamma R-mediated phagocytosis, natural killer cell-mediated cytotoxicity, B cell receptor signaling pathway, NOD like receptor signaling pathway, and T cell receptor signaling pathway, had higher enrichment scores in the ICD high subtype. Therefore, our results indicated that ICD-related genes are tightly associated with the immune-active microenvironment (Fig. 2D).

### Mutations and tumor microenvironment landscape in ICD-high and ICD-low subtypes

**Fig. 3:**
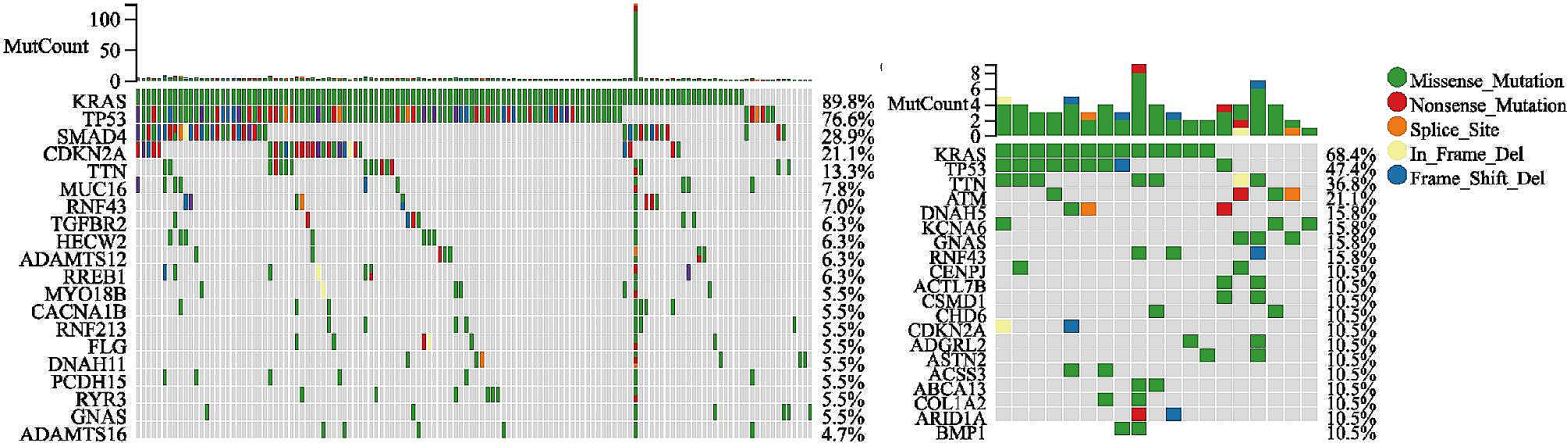
Comparison of the top 20 most frequently mutated genes of two ICD subtypes. (A) ICD-high subtype. (B) ICD-low subtype

Tumor mutation burden is an important biomarker of cancers. Next, we explored the genetic alterations between the two subtypes by using the “maftools” R package. Among them, KRAS and TP53 had the highest mutation frequency, but the mutation rate was much higher in ICD-high subtype (Fig. 3A) (86.5% vs. 68.4% for KRAS and 72.3% vs. 47.4% for TP53). Differentially, following KRAS and TP53, ICD-high subtype showed a high frequency of SMAD4 (25.6%) and CDKN2A (18.6%), but ICD-low subtype (Fig. 3B) demonstrated higher muation frequency of TTN (36.8%) and ATM (21.1%).

### Characterization of the tumor microenvironment (TME) landscape in different subtypes

**Fig. 4:**
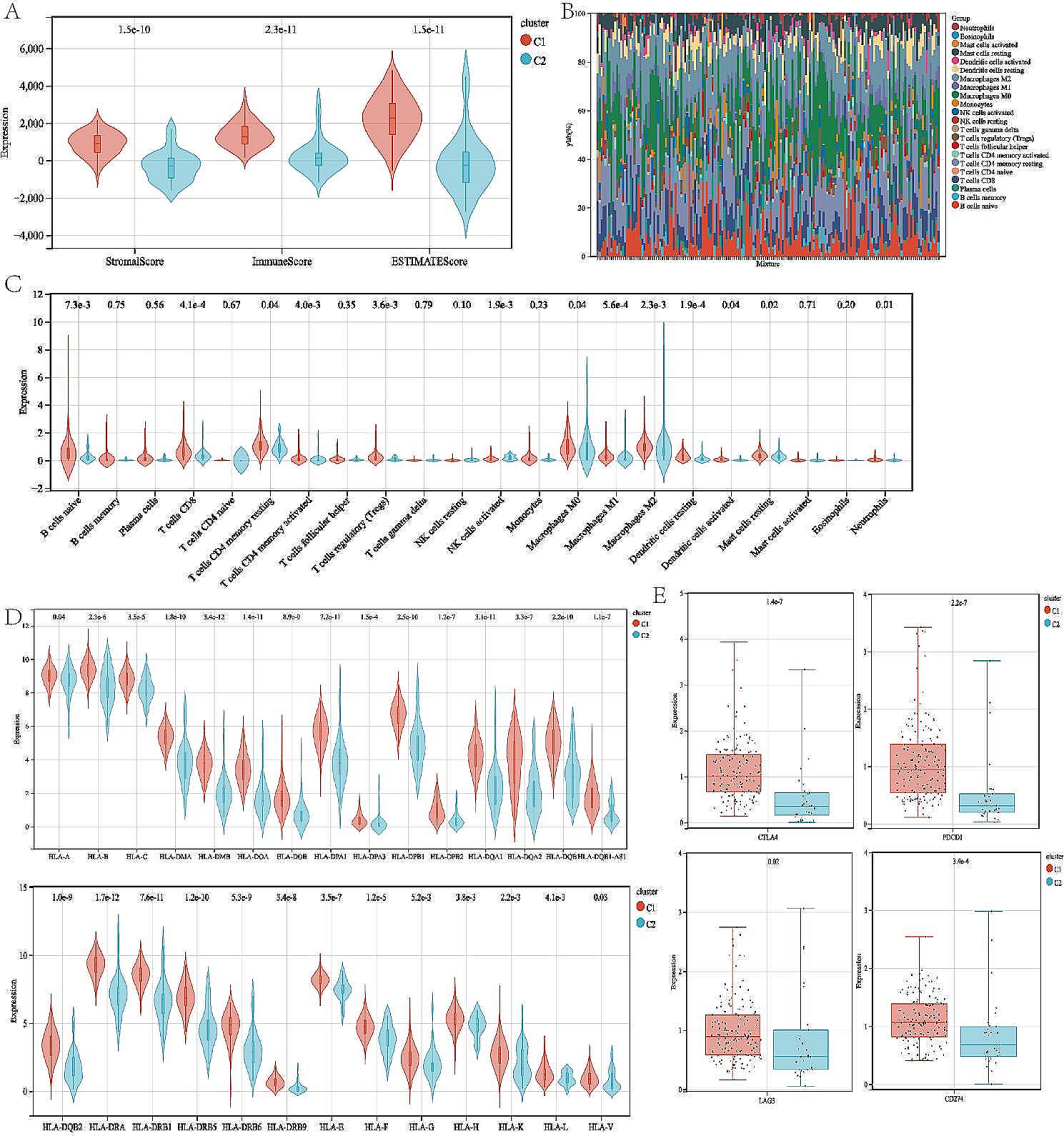
Characterization of the tumor microenvironment (TME) landscape in different subtypes. (A) Violin plots of the stroma score, immune score and ESTIMATE score between different subtypes. (B) Relative proportion of immune infiltration between different subtypes. (C, D) The expression of the immune cells (C) and HLA genes (D) between different subtypes. (E) Box plots of immune checkpoints expression between different subtypes

Given that accumulating evidence strongly indicates that ICD can activate the antitumor immune response and is highly attractive to improve cancer treatment efficacy, we next sort to explore the composition of the tumor microenvironment in different subtypes. By using the ESTIMATE R package, we observed that the stromal score and the immune score were higher in ICD-high subtype (Fig. 4A). More importantly, we found that the ESTIMATE score, which indicated the aggregation of immune or stromal scores in the tumor microenvironment, was statistically higher in ICD-high subtype. Next, we also analyzed the associations between the two subtypes and the immune infiltration of 22 kinds of immune cells utilizing the CIBERSORT approach in conjunction with the LM22 signature matrix (Fig. 4B). We noticed significant variations in the infiltration of some immune cells in different subtypes. The infiltration level of B cells, T cells CD8, T cells CD4 memory resting, T cells CD4 memory activated, Tregs, NK cells activated, Macrophages M0, M1 and M2, dendritic cells activated, and neutrophils were considerably elevated in patients with ICD-high subtype (Fig. 4C).

Moreover, we also evaluated the human leukocyte antigen (HLA) genes between the ICD-high and ICD-low subtypes. We discovered that most HLA genes were deferentially expressed between the two subtypes (Fig. 4D). We further investigated the profile of immune checkpoints in different subtypes. We found that immune checkpoints, including CTLA4, PDCD1 (PD-1), LAG3, and CD274, were significantly upregulated in the ICD-high subtypes, suggesting a potential role of the ICD-related subtypes in immunotherapy (Fig. 4E). In general, our results indicated that the ICD-high subtype in PAAD was associated with immune-hot phenotype.

### Construction and validation of ICD risk model

**Fig. 5:**
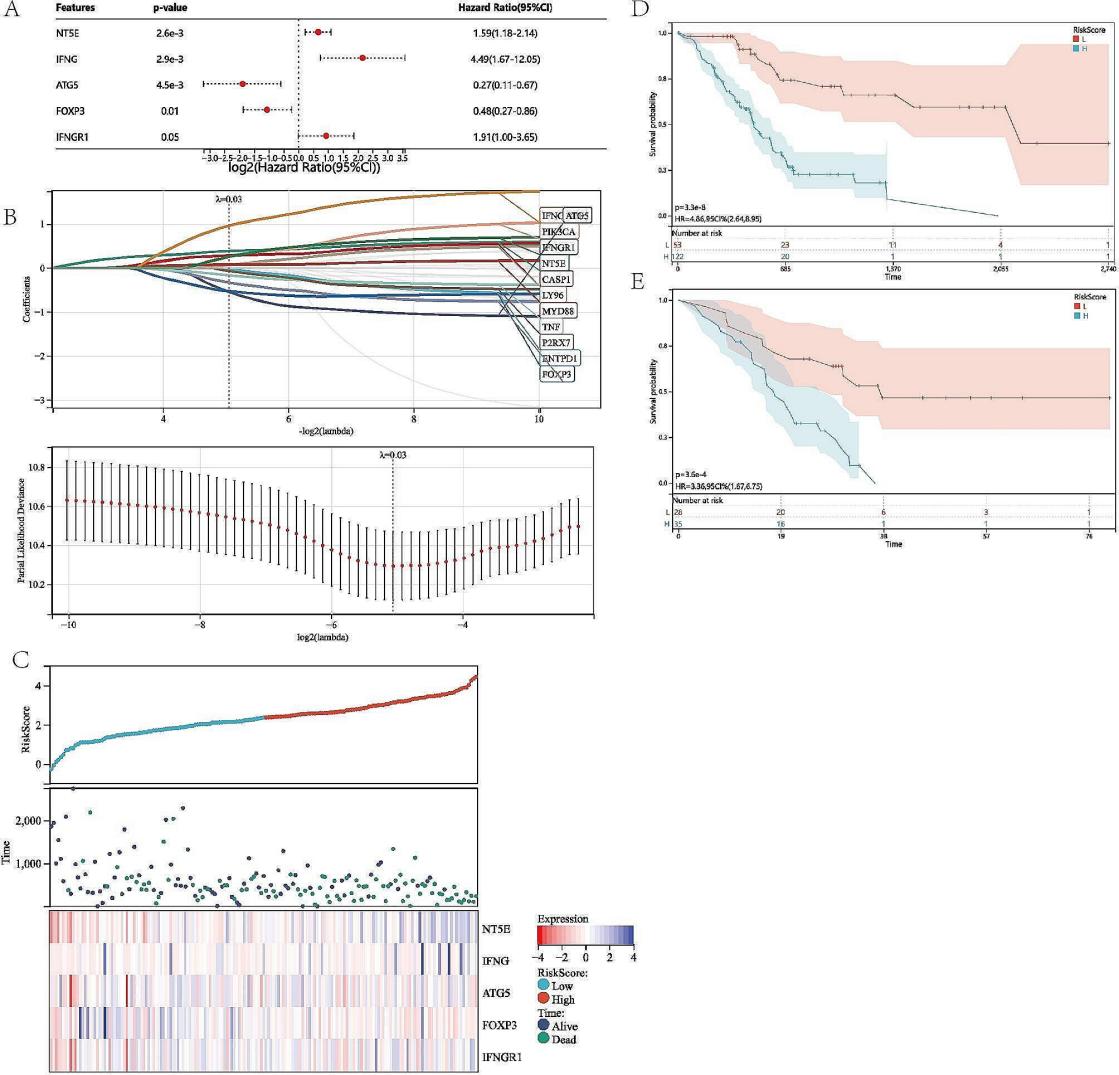
Construction and validation of ICD risk model. (A) Univariate Cox analysis of the prognostic value of the ICD genes in terms of OS. (B) 13 prgnostic ICD-related genes were identified through Lasso Cox analysis. (C) Distribution map of the risk score, individual case survival information, and a clustering heatmap of mRNA expression profiles of the signature genes. (D, and E) Kaplan-Meier analyses of the prognostic significance of the risk model in TCGA and GSE57495 cohort, respectively.

To establish a predictive prognostic model for PAAD patients, we then explored the prognostic-related genes in the PAAD TCGA set. By using the LASSO logistic regression and Cox univariate analysis on the prognostic related genes combined with 10-fold cross validation to narrow the gene scope, we obtained 13 ICD-related genes which were associated with the overall survival of PAAD patients (Fig. 5A, B). The risk score formula was derived to calculate a risk score of each PAAD patient based on the expression level of those genes: Risk Score = (-0.0200)*ENTPD1 +(0.3771)*NT5E+(-0.5485)*ATG5+(0.2836)*PIK3CA+(-0.3363)*IL10+(-0.1699)*TNF+(0.2760)*CASP1+(-0.0697)*P2RX7+(0.0660)*LY96+(0.0787)*MYD88+(-0.5336)*FOXP3+(0.9603)*IFNG+(0.2470)*IFNGR1.

Moreover, we further discovered the relationship between survival status and risk score. We found that the alive status was associated with the low risk score (Fig. 5C). In addition, we also verified the prognostic significance of this risk profile in PAAD by utilizing KM analysis. We found that high risk score was closely associated with worse survival probability (Fig. 5D), which was further verified by similar results in the GEO cohort (Fig. 5E).

### Evaluation of tumor microenvironment in ICD risk models

**Fig. 6:**
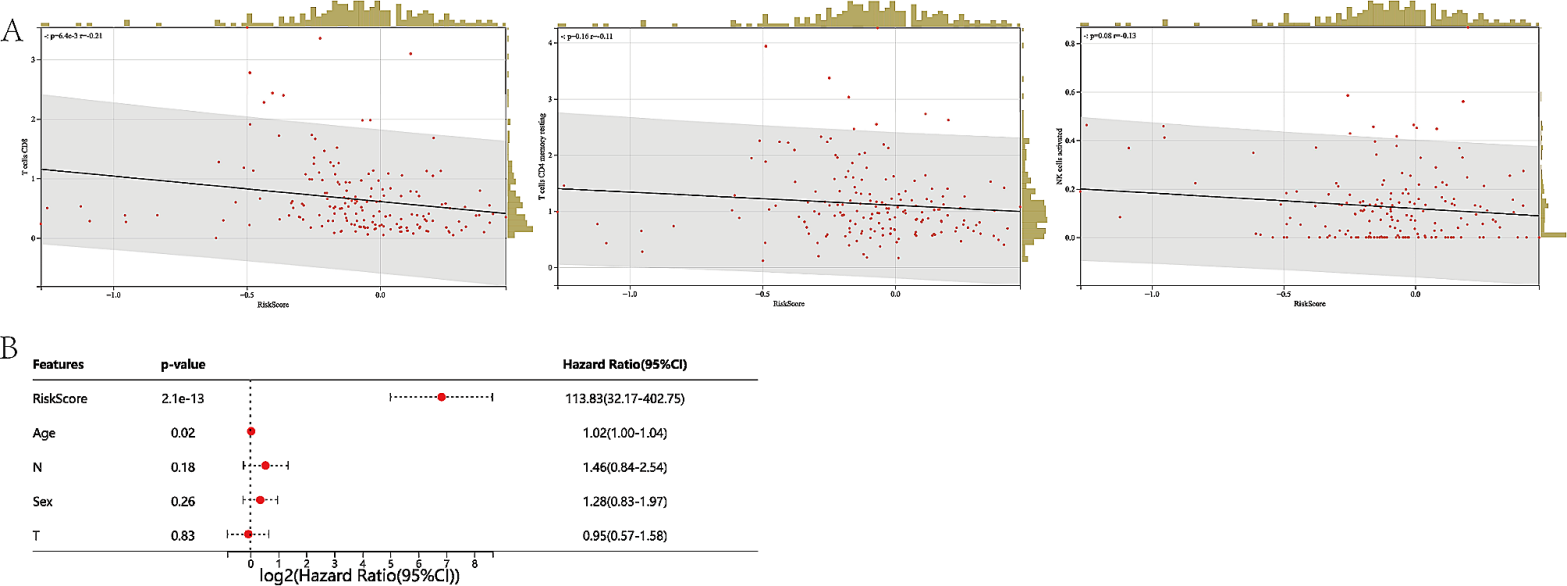
Evaluation of tumor microenvironment in ICD risk models. (A) the correlation of risk score with the infiltration of CD8, activated NK cell, and activated CD4 memory cell. (B) Multivariate Cox analyses of the independent prognostic value of ICD risk signature

To further explore the relationship between ICD risk model and the tumor microenvironment signature, we evaluated the correlation of risk subtypes and immune infiltration cells of PAAD. The results illustrated that the elevated risk score was negatively correlated with the infiltration level of CD4 memory cell, CD8 cell, and NK cell (Fig. 6A).

Next, the independent prognostic value of ICD risk signature, including age, gender, T-stage, and N-stage, were evaluated by multivariate Cox analyses. The results showcased that the ICD-related signature was an independent risk factor for overall survival in patient with PAAD (Fig. 6B).

### Validation of the ICD-related genes for the prognostic signature

**Fig. 7:**
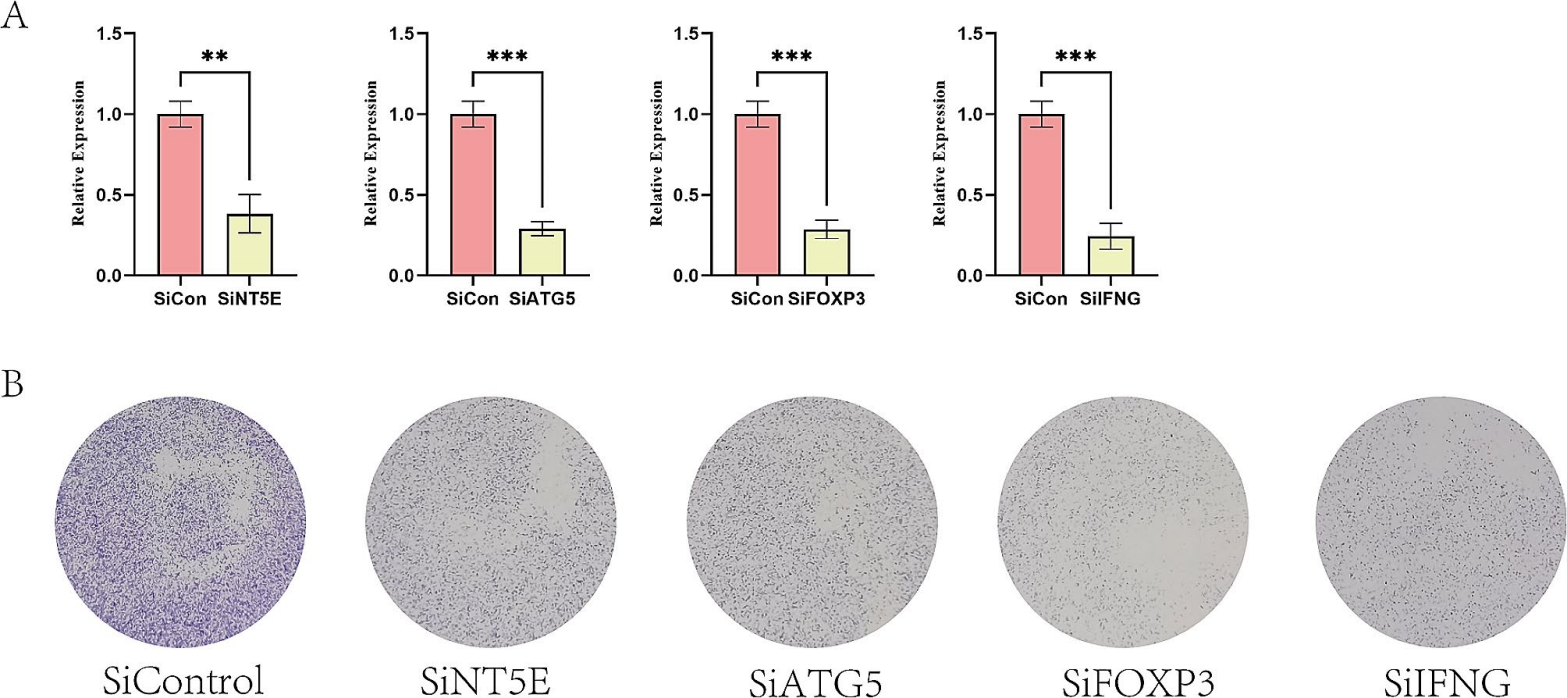
Validation of the ICD-related genes for the prognostic signature. (A) siRNA knockdown efficiencies. (B) The colony formation of PANC-1 cells depletion with NT5E, ATG5, FOXP3, and IFNG

To further confirm the protein expression and the prognostic value of the 13 genes, we selected 4 genes with the highest coefficients (including NT5E, ATG5, FOXP3, and IFNG) in the risk model to perform the colony formation assay. We silenced the expression of each gene through siRNA (Fig. 7A) to further verify the biological functions of those genes. The results of colony formation indicated that depletion of NT5E, ATG5, FOXP3, and IFNG inhibited the colony formation ability of PANC-1 cells (Fig. 7B).

## Discussion

ICD is recognized as a distinct form of regulated cell death that can be induced by different cancer treatments, such as radiotherapy [16], photodynamic therapy [17], and chemotherapeutics [18]. During the process of ICD, specific molecules known as danger-associated molecular patterns (DAMPs) are released, including calreticulin (CRT), high mobility group box 1 (HMGB1), adenosine-5’-triphosphate (ATP), and heat shock proteins (HSPs). These released DAMPs trigger an antigen-specific immune response against a wide range of solid tumors [19].

In our study, we constructed an ICD-related signature and demonstrated its close association with the prognosis and tumor microenvironment of PAAD. We employed the consensus clustering algorithm to classify PAAD patients in the TCGA dataset based on the expression of ICD-related genes. We observed significant differences in clinical features between the two identified clusters. High expression of ICD-related genes was correlated with improved clinical outcomes and increased infiltration of immune cells. Additionally, we identified 13 ICD-related genes with prognostic value in the signature, enabling the stratification of PAAD patients into high-risk and low-risk cohorts. Kaplan-Meier survival analysis with log-rank test further confirmed that patients in the low-risk group exhibited significantly better overall survival, suggesting that this risk signature may serve as an independent prognostic indicator. Furthermore, we selected 4 genes with the highest coefficients (including NT5E, ATG5, FOXP3, and IFNG) to perform the colony formation assay. The results indicated that depletion of those genes inhibited the colony formation ability.

Immunotherapy has transformed cancer treatment by leveraging the immune system to target tumor cells and stimulate specific antitumor immune responses [20]. Despite its advancements, the effectiveness of immunotherapy has been hampered by the immunosuppressive tumor microenvironment [21]. ICD, a type of cell demise, augments the adaptive immune response against tumor cells [22]. During ICD, tumor antigens become accessible to antigen-presenting cells, and damage-associated molecular patterns (DAMPs) are released, facilitating dendritic cell maturation [23], This process leads to the activation of T cells and the infiltration of cytotoxic T cells [24], culminating in robust antitumor immune responses [25]. In line with these findings, our study utilized consensus clustering to identify two distinct ICD clusters. Remarkably, the ICD-high group exhibited a close association with an immune-hot phenotype.

Tumor mutation burden stands as a crucial biomarker in cancer assessment. Pancreatic adenocarcinoma (PAAD) exhibits a distinctive profile characterized by a limited number of recurrent mutations in pivotal oncogenes and tumor suppressor genes, mutations that intricately correlate with disease progression [26]. Among the notable mutations, KRAS, TP53, CDKN2A, and SMAD4 represent the canonical quartet. The oncogenic KRAS mutation emerges as a primary event in pancreatic cancer pathogenesis [27]. TP53 mutations, frequently leading to gain-of-oncogenic activities, are closely tied to invasive and metastatic phenotypes [28]. CDKN2A, responsible for encoding a crucial cell-cycle regulator, prominently emerges as the most frequently altered tumor suppressor gene. Meanwhile, SMAD4, another tumor suppressor gene in PAAD, assumes a pivotal role in mediating downstream signaling of the TGFβ receptor [29]. Notably, somatic mutation analysis across different risk groups reveals a higher likelihood of mutations in KRAS, TP53, CDKN2A, and SMAD4 in ICD-high subtypes (Cluster 1). Previous studies have demonstrated that mutations in CDKN2A, TP53, and SMAD4 are associated with poorer survival and the development of invasive PAAD [30]. This aligns with the observed worse prognosis in our ICD-high subtypes, reinforcing the clinical relevance of these mutations in shaping the disease course.

In summary, our study established a novel classification for PAAD based on the expression of ICD-related genes, and demonstrated significant differences in survival, clinicopathologic features, and immune status between the two clusters. Furthermore, we developed a novel ICD-related gene signature that can predict the outcome of PAAD patients. Overall, these findings provide potential therapeutic strategies against PAAD.

## Data Availability

The datasets used in this study can be found in the GEO database (GSE57495, https://www.ncbi.nlm.nih.gov/geo/query/acc.cgi?acc=GSE57495), and TCGA database (https://portal.gdc.cancer.gov/projects/TCGA-PAAD).
